# Do regulated resident working hours affect medical graduate education? Trends in the American psychiatry board pass rates pre- and post-2003 duty hours regulations^†^

**DOI:** 10.1192/pb.bp.113.046292

**Published:** 2014-12

**Authors:** Gaurav Jain, Kristina Dzara, Mir Nadeem Mazhar, Manisha Punwani

**Affiliations:** 1 Southern Illinois University School of Medicine, Springfield, USA; 2 Yale University School of Medicine, New Haven, USA; 3 Queen’s University, Kingston, Ontario, Canada; 4 University of California San Francisco, USA

## Abstract

**Aims and method** To assess trends of the American Board of Psychiatry and Neurology examination pass rates before and after the 2003 duty hours regulations (DHR). We obtained the pass rates for part I and II for years 2000–2010. Data were divided pre-DHR (2000–2003) and post-DHR (2007–2010).

**Results** During the pre-DHR period, first- and multiple-attempt group pass rates were 80.7% and 39.0% which changed in the post-DHR period to 89.7% and 39.1% respectively. Similarly for the part II exam, the pre-DHR first- and multiple-attempt group pass rates were 60.2% and 43.5% respectively, which increased to 78.7% and 53.8%, among the post-DHR group. Overall, there was a significant increase in the first-attempt candidates pass rates for parts I and II, whereas multiple-attempt candidates did not benefit as strongly.

**Clinical implications** The results suggest that the 2003 DHR may have had a positive impact on examination-based medical knowledge in psychiatry.

In July 2003, the US Accreditation Council for Graduate Medical Education (ACGME) initially mandated nationwide duty hours regulations (DHR) on resident work hours; this lasted until June 2011, when additional rule changes were implemented.^1,2^ While the aim of these changes was to improve working conditions and patient safety, the medical profession has raised concerns about potential negative effects on graduate medical education (GME).^3–5^ Most published studies were subjective and single-institution survey-based, with small sample sizes.^6–8^ Only a few utilised objective measures (such as operative case-loads, standardised in-training examination scores, or the board pass rates at a single or multiple institutions) to examine the impact of the 2003 DHR on the quality of GME and all of them were from surgical specialties.^6–8^ The literature suggests that the 2003 DHR have an influence on three main groups: trainees, faculty and patients.^7,9,10^ Generally, trainees’ perceptions about the effect of the 2003 DHR on their educational experience were neutral to somewhat positive,^5,11,12^ contrary to training directors or faculty members who had somewhat negative to neutral opinions about the effects of DHR on trainee education.^5,7^ Meanwhile, the 2003 DHR have had varying effects on patient outcomes, depending on the indicator, however, there is some evidence of a decrease in mortality for medical and surgical patients.^6,8^ There is no clear consensus regarding the influence of the 2003 DHR on objective GME outcomes in the USA.^6,8–10^

The lack of evidence regarding the effect of the 2003 DHR on GME quality is even more limited in the field of psychiatry.^13^ One published study used a debate format to report opinions of three authors.^14^ The only national survey-based study focusing on psychiatry programmes’ adherence to the 2003 DHR did not assess its impact on GME.^15^ Another multicentre survey-based study examined the effect of the 2011 duty hour changes on the GME in several specialties, including psychiatry, and concluded that it would have a negative impact on GME quality.^16^ All studies also noted that additional research is required to understand the impact of duty hour changes on the GME in psychiatry and other specialties.^14–16^

Until 2010, the American Board of Psychiatry and Neurology (ABPN) administered the psychiatry board examination in two parts.^17^ Part I assessed the knowledge through a 420-item, multiple-choice examination administered via computer for 8 h. Part II (oral) assessed practical interview and clinical skills. It included the examination of simulated patients under the observation of one or more examiners. The manner of examining patients and the subsequent reasoning and deductions constituted an important part of the examination. Knowledge of basic science principles, special diagnostic procedures, management recommendations and assessment of risk were also essential aspects of the examination, which focused on evaluation of clinical skills. For 2000 through 2010, the psychiatry board certification examination was unique as it administered the oral board with a simulated patient section.^18^ A major outcome measure assessing the quality and competency of trained physicians is their ‘board pass’ status. To the best of our knowledge, no study has assessed the trend of the US national board pass rate before and after the 2003 DHR and discussed the possible reasons of change, if any.

## Method

We requested psychiatry board pass data (part I and part II) from the ABPN for years 2000 through 2010 matched with the candidate’s age, gender, medical school location, type of residency programme (university-based, university-affiliated or community-based) and number of attempts. In 2011 a major change occurred, with the combination of both parts into a single computer-based examination. Thus, we excluded 2011 and onwards from our analyses.

However, due to certain limitations ABPN was only able to provide limited year-wise aggregate data for the psychiatry boards. For each year from 2000 through 2010, we obtained the number of total and first-attempt candidates who took the exam and the number who passed for parts I and II. To assess the effect of the 2003 DHR change, we divided the data into two groups: pre-DHR (2000–2003), before the regulations went into effect, and post-DHR (2007–2010), as 2007 was the first year when graduates fully trained under the 2003 DHR modification began to take boards. To analyse potential variability in the effect of the 2003 DHR changes by attempt type (first- *v*. multiple-attempt candidates), we calculated the number of candidates who attempted the test more than one time, as well as their pass rates. We defined this group as multiple-attempt candidates.

We divided the data into six subgroups for analysis: (1) part I total; (2) part I first attempt; (3) part I multiple attempt; (4) part II total; (5) part II first attempt; and (6) part II multiple attempt. We analysed the differences pre- and post-DHR change within these subgroups, using SPSS on Windows. Chi-squared tests determined whether there were significant differences between the pre- and post-DHR subgroups. Finally, for each of the six groups, we calculated odds ratios to determine the likelihood of those in the post-DHR group passing the boards, compared with those who took boards prior to the DHR change. We also calculated 95% confidence intervals.

## Results

Table 1 displays the number of total and first-attempt candidates, number passed and pass rates of the ABPN’s psychiatry part I and II examinations, over time, for years 2000–2010. The calculated multiple-attempt candidates pass rates are also displayed. There is a trend of a gradual increase in the total pass rates and first-attempt pass rates for both part I and part II. Multiple-attempt pass rates were generally stable for part I, whereas there is an upward trend for part II.

For part I, the average total, first-attempt and multiple-attempt pass rates before the 2003 DHR change (2000–2003) were 64.0%, 80.7% and 39.0% respectively. Post-DHR change (2007–2010), these increased to 76.8%, 89.7% and 39.1%. Similarly for part II, 53.5%, 60.2% and 43.5% of the total, first-attempt and multiple-attempt candidates passed pre-DHR respectively. Post-DHR change, these rates increased to 71.8%, 78.7% and 53.8%.

Table 2 presents the results of the analyses comparing total, first-attempt and multiple-attempt pass rates between the pre- and post-DHR change groups. For part I, compared with those who took the tests pre-DHR changes, odds ratios suggested a total increase of 86.5% (95% CI 1.734–2.005) in the likelihood of passing. First-attempt candidates increased their odds of passing by 109.3% (95% CI 1.864–2.350). However, multiple-attempt candidates did not have a significant increase (*P* = 0.945).

Post-DHR change, the overall odds of passing part II increased by 120.3% (95% CI 2.056–2.360). First-attempt candidates increased their odds by 143.5% (95% CI 2.226–2.663), and multiple-attempt candidates’ odds increased by 51.4% (95% CI 1.351–1.697).

## Discussion

Although a large amount of discourse surrounds the effect of DHR changes on the quality of GME, few studies have utilised objective measures to evaluate its impact. Three review articles noted that ten studies assessed the impact of the 2003 DHR changes on standardised examination scores:^6,8^ nine from surgical specialties and one from obstetrics and gynaecology. Two reported an improvement in test scores after the DHR, and two reported a decrease; five showed no change in scores. Only one study compared American Board of Surgery pass rates pre- and post-2003 DHR, although comparisons were limited to 17 sites in New England.^19^ Our study found improved pass rates in the post-2003 DHR cohort, compared with the pre-DHR cohort, as measured through national psychiatry board pass rates in the USA. In addition, first-time test takers, who likely recently graduated, appear to have improved performance, which may in part be due to these changes. This finding is supported by the marked increase in first-attempt candidates’ pass rates for part I, compared with the multiple-attempt candidates who appear to receive little benefit from the DHR changes. This is unique and suggests that DHR changes may have helped residents improve their examination-based medical knowledge.

**Table 1:** American Board of Psychiatry and Neurology pass rates for part I and part II examinations (2000-2010)^a^

	Candidates *n*	Passed *n* (%)	FAC *n*	FAC passed *n* (%)	MAC passed *n* (%)^b^
Part I					
2010	1840	1415 (76.9)	1412	1253 (88.7)	37.9
2009	1795	1421 (79.2)	1400	1264 (90.3)	39.7
2008	1575	1229 (78.0)	1139	1057 (92.8)	39.4
2007	1734	1269 (73.2)	1222	1068 (87.4)	39.3
2006	1826	1329 (72.8)	1236	1056 (85.4)	46.3
2005	1816	1281 (70.5)	1196	996 (83.3)	46.0
2004	1680	1137 (67.7)	1119	900 (80.4)	42.2
2003	1815	1282 (70.6)	1186	1002 (84.5)	44.5
2002	1812	1203 (66.4)	1098	896 (81.6)	43.0
2001	1960	1176 (60.0)	1149	912 (79.4)	32.6
2000	2105	1261 (59.9)	1180	912 (77.3)	37.7
Part II					
2010	1761	1307 (74.2)	1319	1043 (79.1)	59.7
2009	1815	1311 (72.2)	1347	1070 (79.4)	51.5
2008	1657	1192 (71.9)	1172	931 (79.4)	53.8
2007	1750	1203 (68.7)	1208	926 (76.7)	51.1
2006	1766	1199 (67.9)	1148	870 (75.8)	53.2
2005	1806	1129 (62.5)	991	705 (71.1)	52.0
2004	2146	1157 (53.9)	1260	789 (62.6)	41.5
2003	1996	1041 (52.2)	1104	650 (58.9)	43.8
2002	1881	967 (51.4)	1138	660 (58.0)	41.3
2001	1867	997 (53.4)	1123	689 (61.4)	41.4
2000	1922	1097 (57.1)	1213	759 (62.6)	47.7

FAC, first-attempt candidates; MAC, multiple-attempt candidates.

a. Source: American Board of Psychiatry and Neurology (http://www.abpn.com/downloads/misc_publications/pc_2011_AADPRT-Presentation_final.pdf and http://www.abpn.com/cert_statistics.html).

b. Multiple attempt candidates include those who have taken the test more than once. $\text{MAC pass rate}=\frac{\left(\text{total passed}-\text{FAC passed}\right)\;100}{\left(\text{total candidates}-\text{FAC}\right)}$

### Limitations

There are several limitations of our study. First, there were significant changes in the examination style and evaluation.^20^ In April 2005, the part II scoring system changed and in May 2006 the part II test format changed for the audiovisual (non-patient) section. Further, in 2008 the part I test administration date changed from fall to summer. Each of these may have had an independent effect on the pass rates.

Second, in July 2001 the ACGME introduced the teaching and assessment of core competencies as a part of

**Table 2:** Pass rate comparison between pre- and post-2003 duty hour restriction periods

	Part I pass rates			Part II pass rates		
	Total	FAC	MAC^a^	Total	FAC	MAC^a^
2000-2003	64.0%	80.7%	39.0%	53.5%	60.2%	43.5%
2007-2010	76.8%	89.7%	39.1%	71.8%	78.7%	53.8%
*P*	***	***	*P* = 0.945	***	***	***
Odds ratio	1.865	2.093	1.004	2.203	2.435	1.514
(95% CI)	(1.734-2.005)	(1.864-2.350)	(0.891-1.132)	(2.056-2.360)	(2.226-2.663)	(1.351-1.697)

FAC, first-attempt candidates; MAC, multiple-attempt candidates.

a. Multiple-attempt candidates include those who have taken the test more than once.

*** *P*<0.0001.

common programme requirements.^21^ The ACGME is emphasising phase-in and progressive improvement in the implementation of these competencies in four phases;^21^ in July 2011, the implementation entered into its final phase. This progressive change may have influenced pass rates. Overall, these two changes were made to streamline the process of training and testing, hence they would have likely contributed to the positive change. Specifically, the changes to the psychiatry part II examination may have had a positive impact on pass rates. However, our results suggested that there was a stronger impact on the likelihood of passing part II among the first-attempt candidates than multiple-attempt candidates. Therefore, the difference between pre- and post-DHR part II pass rates cannot be accounted for solely by the examination system changes.

Third, because of data limitations, we were unable to compute full statistical models controlling for all variables which may have had an effect on pass rates. These variables include but are not limited to age, gender, medical school location, United States Medical Licensing Examination scores, type of residency programme (university-based, university-affiliated or community-based), prior training, and utilisation of board preparation courses.

Despite these limitations, the total and first-attempt pass rates suggest an overall positive trend, which may be partly attributed to the DHR changes. Future studies to eliminate these limitations and including a new post-2011 DHR group may be helpful in ascertaining the contribution of the DHR changes in a candidate’s board examination result.

This is the first study which analysed national board pass rates in an attempt to assess the impact of the 2003 DHR changes on the examination-based medical knowledge of board-eligible candidates. Overall, we found a significant trend of increases in the total and first-attempt pass rates for both part I and part II of the ABPN psychiatry boards. However, due to limitations we were unable to determine the amount of contribution made by the DHR change in this trend. As the specialty of psychiatry has not been at the forefront of the debate over residents’ work hours, our findings may not be generalisable to other specialties as well. Despite the shortcomings, this study contributes to the literature surrounding the effect of the DHR changes on GME and suggests that the changes may have made a positive contribution to this aspect of resident training in psychiatry.
